# Nintedanib treatment for pulmonary fibrosis after coronavirus disease 2019

**DOI:** 10.1002/rcr2.744

**Published:** 2021-03-30

**Authors:** Hiroaki Ogata, Taisuke Nakagawa, Soichiro Sakoda, Akiko Ishimatsu, Kazuhito Taguchi, Masako Kadowaki, Atsushi Moriwaki, Makoto Yoshida

**Affiliations:** ^1^ Department of Respiratory Medicine National Hospital Organization Fukuoka National Hospital Fukuoka Japan; ^2^ Department of Infectious Diseases National Hospital Organization Fukuoka National Hospital Fukuoka Japan

**Keywords:** Acute respiratory distress syndrome, coronavirus disease 2019, nintedanib, pulmonary fibrosis, severe acute respiratory syndrome coronavirus 2

## Abstract

A 78‐year‐old Japanese woman with no smoking history suffered from near‐fatal coronavirus disease 2019 (COVID‐19) requiring four‐week invasive mechanical ventilation, with subsequent radiological features of pulmonary fibrosis. Although methylprednisolone gradually improved her respiratory condition, her oxygenation and exercise tolerance had drastically deteriorated, necessitating high‐flow nasal cannula oxygen therapy. In parallel with tapering systemic steroid, the patient was treated with nintedanib. Three months later, the patient was able to walk with a walking aid using oxygen at 4 L/min. The present case is an indication that nintedanib might provide a novel therapeutic approach for managing post‐COVID‐19 fibrosis, although further studies are warranted.

## Graphical abstract

Pulmonary fibrosis after coronavirus disease 2019 (COVID‐19) recovery is becoming an emerging threat to public health worldwide. Given the present case, nintedanib might provide a novel therapeutic approach for managing post‐COVID‐19 fibrosis, although further studies are warranted.

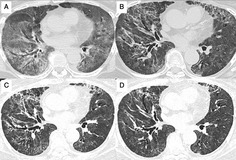

## Introduction

The novel coronavirus disease 2019 (COVID‐19), caused by severe acute respiratory syndrome (SARS) coronavirus 2 (SARS‐CoV‐2), became a global pandemic within a short period. In many fatal or near‐fatal cases, the development of pulmonary fibrosis secondary to acute respiratory distress syndrome (ARDS) is inevitable [1, 2]. Taking into account the second wave of COVID‐19 outbreak since autumn 2020, pulmonary fibrosis after COVID‐19 recovery could be an emerging threat to, and a burden on, public health worldwide in the near future.

Nintedanib is an antifibrotic drug, inhibiting fibrogenesis across a wide range of pulmonary disorders, including connective tissue‐associated interstitial lung diseases [3]. As profibrotic pathways in post‐COVID‐19 pulmonary fibrosis have been considered to resemble those in autoimmune interstitial lung diseases, nintedanib might have beneficial effects in the management of the pulmonary consequences of COVID‐19 [1]. However, there has been no clinical report on the effect of nintedanib on persistent pulmonary impairment as a sequela of COVID‐19. We now present a case of diffuse pulmonary fibrosis persisting after near‐fatal COVID‐19, with the patient being treated with nintedanib in parallel with tapering systemic corticosteroids.

## Case Report

**Figure 1:**
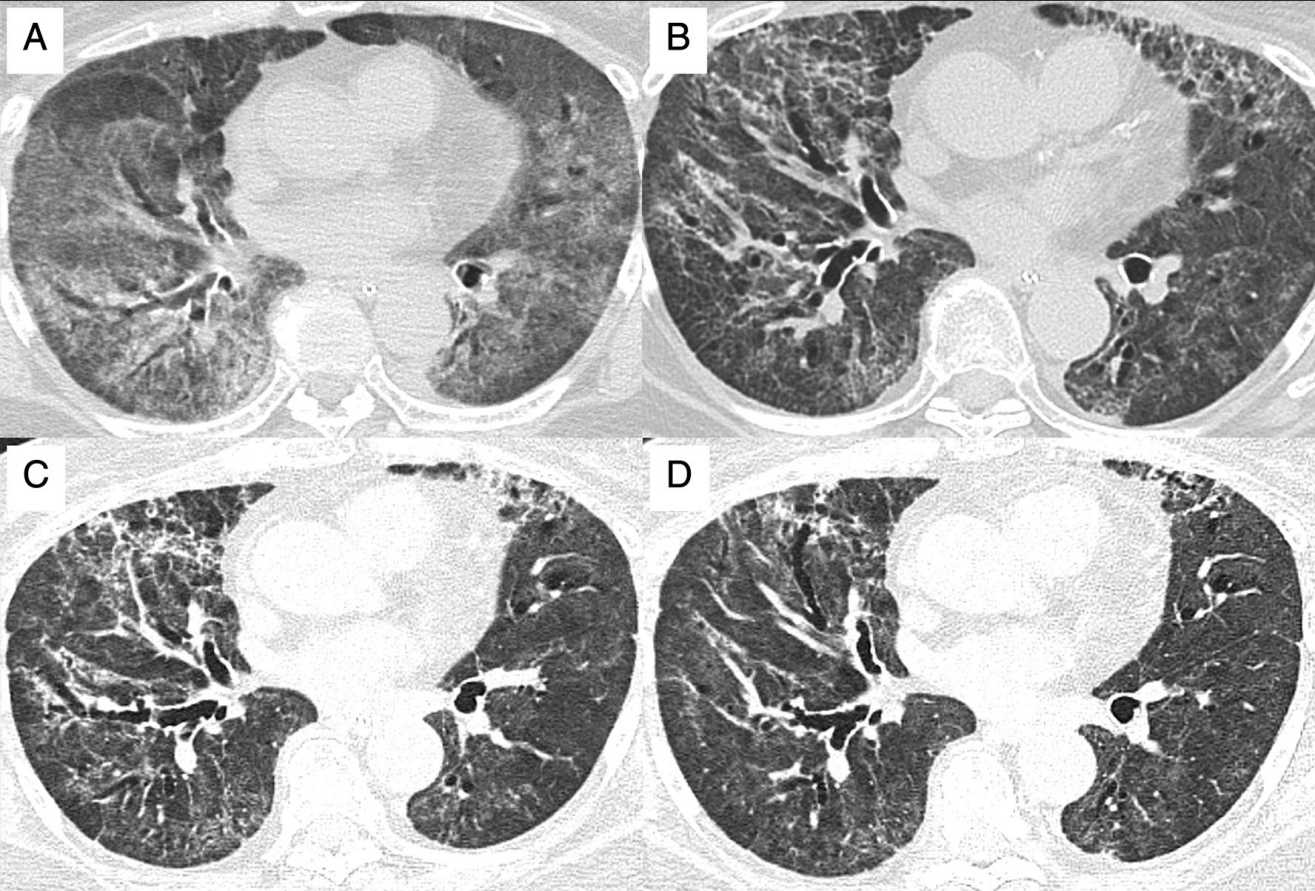
Computed tomography images of the chest (A) when requiring mechanical ventilation; (B) two weeks after extubation, requiring high‐flow nasal cannula oxygen therapy and nasogastric tube feeding; (C) when initiating nintedanib therapy; and (D) two months after the implementation of nintedanib treatment.

A 78‐year‐old Japanese woman with no smoking history suffered from COVID‐19‐induced ARDS (Fig. 1A), which required multidisciplinary management in the intensive care unit, including invasive mechanical ventilation. She had no underlying disease except for hypertension. The patient was initially treated with the combination therapy of remdesivir, dexamethasone (6 mg/day), and heparin for 10 days, with no beneficial effect on respiratory failure; 14 days after tracheal intubation, her respiratory condition worsened. Subsequent use of methylprednisolone at a dose of 80 mg/day gradually improved her respiratory condition such that she could be extubated on day 28 after intubation (Fig. 1B). However, residual pulmonary impairment had drastically deteriorated her oxygenation and exercise tolerance; it was hard for her even to eat or swallow, necessitating nasogastric tube feeding as well as high‐flow nasal cannula oxygen therapy. Twenty‐five days after extubation, the patient was transferred to our hospital for further management.

Concurrently, with decreasing systemic steroid doses, the patient underwent pulmonary rehabilitation, which gently relieved her dyspnoea and led to removal of the nasogastric tube. Regarding the findings from computed tomography of the chest, diffuse ground‐glass opacities of the lung were passably attenuated (more than half of the opacities were diminished or improved), whereas diffuse reticular changes with traction bronchiectasis appeared, suggesting the development of lung fibrosis (Fig. 1C). On day 12 after transfer, that is, day 65 after the initiation of mechanical ventilation, the patient was started on oral treatment with nintedanib at a dose of 300 mg/day. Although the serum level of alanine aminotransferase became elevated as an adverse event, it was safely managed with nintedanib dose reduction to 200 mg/day and the use of ursodeoxycholic acid. No other side effects of nintedanib were observed. Pulmonary function tests were performed six weeks after the initiation of nintedanib therapy, and the patient demonstrated restrictive ventilatory impairment (vital capacity 1.27 L, vital capacity per predicted 61.7%). After three months of rehabilitation with nintedanib and ongoing tapering of prednisolone to 10 mg on alternate day, the respiratory condition and exercise tolerance improved; the patient was able to walk with a walking aid using oxygen at 4 L/min. Ground‐glass opacities of the lung were mildly attenuated without further progression of pulmonary fibrosis (Fig. 1D), while the systemic steroid was tapered to 10 mg every other day of treatment with prednisolone. The patient continues rehabilitation in the hope of hospital discharge to home, alongside the tapering of prednisolone to full withdrawal in a few months.

## Discussion

Although the long‐term consequences of COVID‐19 remain largely unknown, there has been considerable speculation that patients with COVID‐19 pneumonia, especially severe or critical cases, are as likely to develop pulmonary fibrosis as SARS and Middle East respiratory syndrome (MERS) [1, 2]. Considering the rapidly evolving and expanding pandemic of COVID‐19, the burden of pulmonary fibrosis after COVID‐19 recovery will likely be substantial and become a major health concern worldwide [4]. To date, there has been no therapeutic intervention preventing or managing the fibrotic outcomes of the lung in COVID‐19. As far as we are aware, the present case is the first reported post‐COVID‐19 pulmonary fibrosis treated with nintedanib.

Nintedanib is a tyrosine kinase inhibitor with selectivity for the vascular endothelial growth factor (VEGF), platelet‐derived growth factor (PDGF), and fibroblast growth factor (FGF) receptors. It has been demonstrated to be beneficial for not only idiopathic pulmonary fibrosis, but also various other forms of progressive pulmonary fibrosis [3]. The plasma concentration of VEGF, PDGF, and FGF was found to increase among COVID‐19 patients [5], leading to expectation of the effectiveness of nintedanib. In addition, this agent has been shown to decrease the expression of IL‐1 and IL‐6, which play a central role in COVID‐19 cytokine storm leading to fibrogenesis in lung [1]. Among the wide range of progressive fibrosing interstitial lung diseases, nintedanib therapy might inhibit the pathogenetic profibrotic pathways induced by SARS‐CoV‐2.

As nintedanib is commercially available only in an oral capsule formulation and therefore cannot be crushed [6], its administration was postponed for two months in the current case due to endotracheal intubation and nasogastric tube feeding. Given the overexpression of proinflammatory cytokines during acute lung injury by SARS‐CoV‐2 [5], earlier intervention with nintedanib might have led to somewhat more favourable effects on fibrotic consequences. In the present case, radiological changes of pulmonary fibrosis stabilized with gradual resolution of ground‐glass opacities, while being treated with nintedanib and systemic steroid. Further studies are warranted to establish an appropriate strategy for preventing and managing pulmonary fibrosis with COVID‐19.

In conclusion, nintedanib might provide a novel therapeutic approach for managing COVID‐19‐induced pulmonary fibrosis. Given the scale of the COVID‐19 pandemic and the number of patients requiring invasive ventilation worldwide, post‐COVID‐19 pulmonary fibrosis is becoming a lethal threat to global health. To deal with this emerging issue, trials evaluating the efficacy and safety of nintedanib against COVID‐19‐related fibrotic complications of lung are necessary.

### Disclosure Statement

Appropriate written informed consent was obtained for publication of this case report and accompanying images.

### Author Contribution Statement

Hiroaki Ogata contributed to the conception of the work, the acquisition and interpretation of data for the work, and drafting of the manuscript. Taisuke Nakagawa, Soichiro Sakoda, Akiko Ishimatsu, Kazuhito Taguchi, Masako Kadowaki, and Atsushi Moriwaki contributed to the interpretation of data for the work and revision of the manuscript. Makoto Yoshida contributed to the conception of the work, interpretation of the data, and revision of the manuscript. All authors critically reviewed the manuscript and approved the final version.
